# Non-surgical treatment of oblique diaphyseal fractures of the fourth and fifth metacarpals in a professional athlete: A case report

**DOI:** 10.1016/j.ijscr.2024.109256

**Published:** 2024-01-12

**Authors:** Paolo Boccolari, Filippo Pantaleoni, Danilo Donati, Roberto Tedeschi

**Affiliations:** a Physical Therapy and Rehabilitation Unit, Policlinico di Modena, 41125 Modena; b Department of Biomedical and Neuromotor Sciences, Alma Mater Studiorum, University of Bologna, Bologna, Italy; c Clinical and Experimental Medicine PhD Program, University of Modena and Reggio Emilia, 41121 Modena, Italy

**Keywords:** Metacarpal fractures, Sports injuries, Hand injuries, Conservative treatment, Functional recovery

## Abstract

**Introduction:**

Metacarpal fractures are common sports-related injuries, often requiring tailored treatment strategies, especially in athletes. The management of oblique diaphyseal fractures poses unique challenges due to their inherent instability. This case report discusses a non-surgical approach in treating such fractures in a professional athlete.

**Case presentation:**

A 26-year-old professional soccer player sustained oblique diaphyseal fractures of the fourth and fifth metacarpals during training. Given the athlete's professional demands and the fracture's nature, a conservative treatment was implemented. This included the application of a modified ulnar gutter brace, allowing for immobilization of the metacarpophalangeal joints (MP) while permitting active mobilization of the interphalangeal joints(IP).

**Clinical discussion:**

The non-surgical treatment focused on achieving skeletal stability and maintaining hand function. Despite the complexity of oblique fractures, the conservative approach was successful, enabling the athlete to resume professional activities with minimal risk of fracture displacement. Regular radiographic follow-ups showed no further displacement, highlighting the effective management of such fractures through personalized conservative treatment plans.

**Conclusions:**

This case underscores the viability of conservative treatment for specific metacarpal fractures in athletes. Tailoring the treatment to accommodate the athlete's professional needs and understanding the biomechanical characteristics of the fracture are crucial for successful outcomes. The case also suggests that non-surgical management can be a viable option for certain complex metacarpal fractures, especially in high-demand patients.

## Highlights

• Effective in managing spiral fractures of the fourth and fifth metacarpals.
• Allowed continued play with minimal hindrance, avoiding additional fracture displacement.
• The player resumed competitive matches soon after injury, showcasing treatment practicality in high-demand sports.
• Aligns with current trends in fracture management, emphasizing early mobilization.
• Radiographic follow-ups confirmed stable and proper healing, highlighting the treatment's efficacy.

## 1 Introduction

Metacarpal fractures represent about 18 % of fractures below the elbow and 44 % of all hand fractures, with a significant impact on athletes' performance due to potential long-term absences and residual stiffness [1,2]. The hand's functionality relies on the balance between intrinsic and extrinsic muscles, flexors, and extensors, with metacarpal length playing a crucial role [3]. Fractures can disrupt this balance by altering the longitudinal structure of the bone [4].

These muscles also support the hand's longitudinal and transverse arches. Fractures can disturb these arches, transforming intrinsic and extrinsic muscles into potential destabilizers of the fracture [5]. The primary treatment objective is to restore skeletal stability and length while maintaining function, prioritizing functional restoration over anatomical perfection in radiographic outcomes [6].

Treatment challenges arise from the dichotomy between immobilization for healing and the risk of joint stiffness and tendinous adhesions [7]. In the hand, particularly susceptible to stiffness, this can lead to significant functional impairment [8,9].

Metacarpal fractures are classified as transverse, short and long oblique, and comminuted. Transverse diaphyseal fractures are stable, while oblique fractures are inherently unstable due to their geometry, as outlined by Maureen and Feehan [10,11].

Approximately 80 % of athlete metacarpal fractures are compound or slightly displaced, treatable non-surgically [[12], [13], [14], [15]]. The case report focuses on oblique fractures, typically resulting from torsional injuries. Intrinsic and extrinsic muscles can exacerbate these fractures, leading to skeletal deformities like shortening and malrotation. The second and third metacarpals are less prone to shortening due to the interosseous ligament, which acts as a suspension system [16]. Biomechanical studies have shown significant functional impairment with even minimal shortening [17].

Treatment planning involves radiographic assessment, clinical evaluation for potential deformities [18], and early mobilization with protective orthosis. The orthosis should immobilize the metacarpophalangeal joints at 70°-90° flexion, balancing ligament tension and minimizing intrinsic muscle destabilization. It should allow proximal wrist movement and immediate active interphalangeal joint mobilization. Syndactyly between adjacent fingers can help control malrotation [19].

## 2 Case presentations

**Fig. 1:**
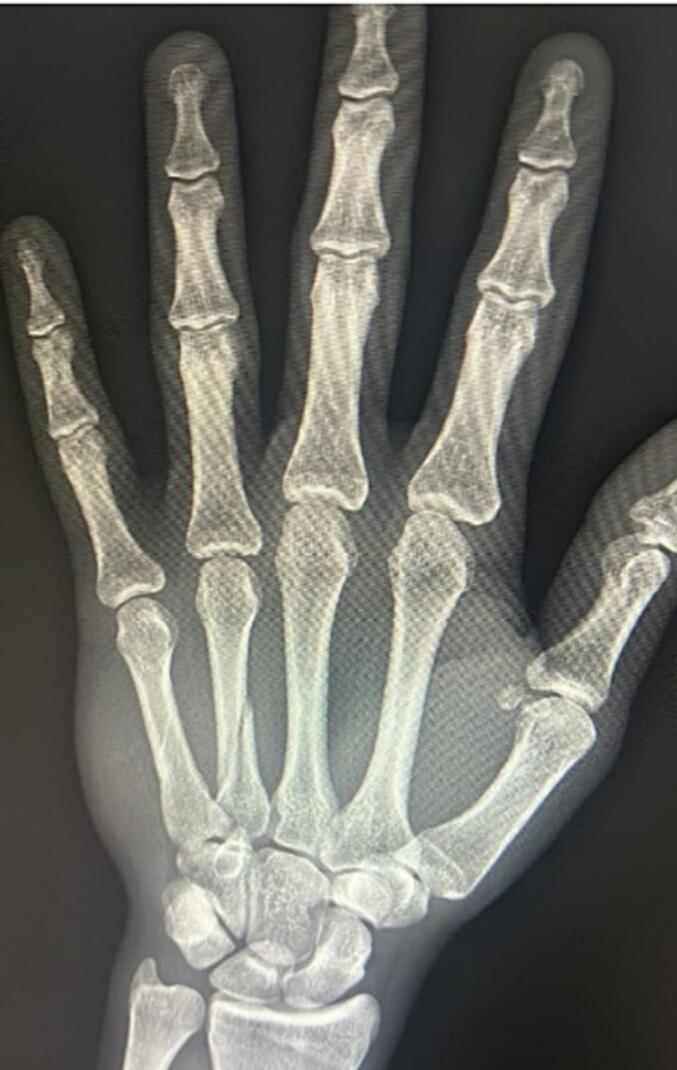
Xray hand.

he subject is a 26-year-old professional soccer player, a midfielder in the Italian Serie A. The dominant hand of the athlete is not specified. The injury occurred during a training session, where the athlete fell and sustained a spiral diaphyseal fracture of the fourth and fifth metacarpals (Fig. 1). Following the injury, the patient was taken to the nearest hospital for appropriate medical examinations, where control radiographs revealed the said dual metacarpal fracture, appearing slightly displaced in two radiographic projections.

At the Hospital hand surgery center, the attending physician re-evaluated the case and the radiographic images. Clinical assessment involved asking the patient to fully flex the fingers to check for malrotations, which were absent. The patient was also asked to extend the fingers to check for any extensor lag in the MP/IP of the fourth and fifth fingers, which was also negative. The radiographic image of slight displacement of both fractures was confirmed.

Literature suggests that conservative treatment is not indicated in the presence of two metacarpal fractures, and the slight displacement initially led the physician to recommend surgical intervention. The case was discussed in a team with another doctor and a hand therapist. Surgery would have allowed solid stabilization of the fracture, enabling the player to return to the field safely three days later for a championship match. An alternative would have been to stabilize the fractures with a modified ulnar gutter brace orthosis for a non-surgical treatment approach. The potential risk of this approach was single or double displacement of the fractures due to the player's exertions during the match. The final decision by the team, along with the team doctor, was for conservative treatment, aware that post-match displacement would necessitate a surgical intervention.

## 3 Clinical findings

Type of Injury: The patient, a 26-year-old professional soccer player, sustained spiral diaphyseal fractures of the fourth and fifth metacarpals during a training session.

Initial Examination: Upon initial medical examination at a nearby hospital, control radiographs revealed a dual metacarpal fracture, with the fractures appearing slightly displaced in the radiographic projections.

Further Evaluation at a Specialized Center: At the Hospital hand surgery center, the attending physician conducted a detailed re-assessment of the case and the radiographic images.

Clinical Assessment for Malrotation: The patient was asked to fully flex the fingers to check for any malrotations. The clinical assessment found no evidence of malrotation in the affected fingers.

Evaluation for Extensor Lag: The patient was also asked to extend the fingers to assess for any extensor lag in the MP and IP joints of the fourth and fifth fingers. This examination also yielded negative results, indicating no extensor lag.

Confirmation of Radiographic Findings: The clinical examination confirmed the radiographic findings of slight displacement of both fractures.

Consideration of Treatment Options: The clinical findings, along with the literature review and team discussions, initially indicated a preference for surgical intervention due to the nature and displacement of the fractures. However, after comprehensive deliberation, a decision was made to opt for a conservative treatment approach with awareness of the potential risks and need for possible future surgical intervention. This case study adheres to the SCARE [20] (Surgical Case Report) guidelines for reporting surgical case studies. The SCARE guidelines aim to enhance the transparency and completeness of reporting surgical cases, providing a structured framework that facilitates accurate communication and assessment of surgical experiences.

### 3.1 Treatment

**Fig. 2:**
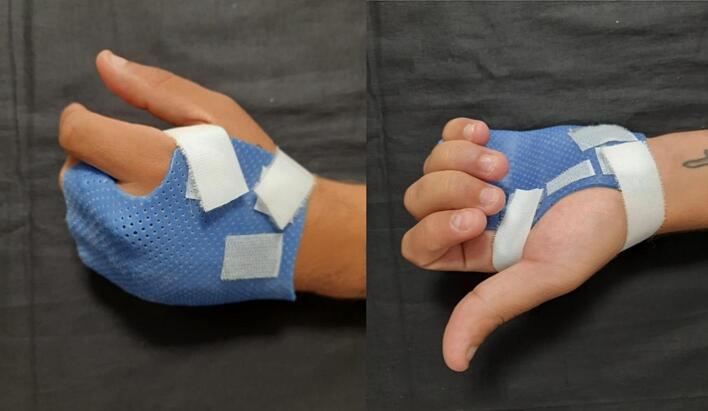
Static ulnar gutter brace orthosis.

The hand therapist crafted a static ulnar gutter brace orthosis, modified to flex the MP of the third, fourth, and fifth fingers at 70°, leaving the second finger outside the brace. This positioning is critical for two reasons: it reduces the destabilizing action of the interosseous muscles on the metacarpals and tensions the intermetacarpal ligament, which acts as a suspension cradle for the distal fracture stumps. The third finger was included in the brace to utilize the tension of the ligament portion between the third and fourth fingers, thereby providing more effective suspension of the fracture stumps. As described in the literature, the modified brace does not include the wrist and the IP, which are actively mobilized immediately as part of the patient's self-treatment at home. Since the player had to participate in both Sunday matches and weekly training sessions, the orthosis was equipped with a removable extension to be used only during gameplay, to protect the fingers outside the brace. This extension is U-shaped, open on the radial side, and attached to the gutter with velcro. The player was also advised to secure the extension with adhesive tape during matches to prevent it from coming off in a collision on the field (Fig. 2).

**Fig. 3:**
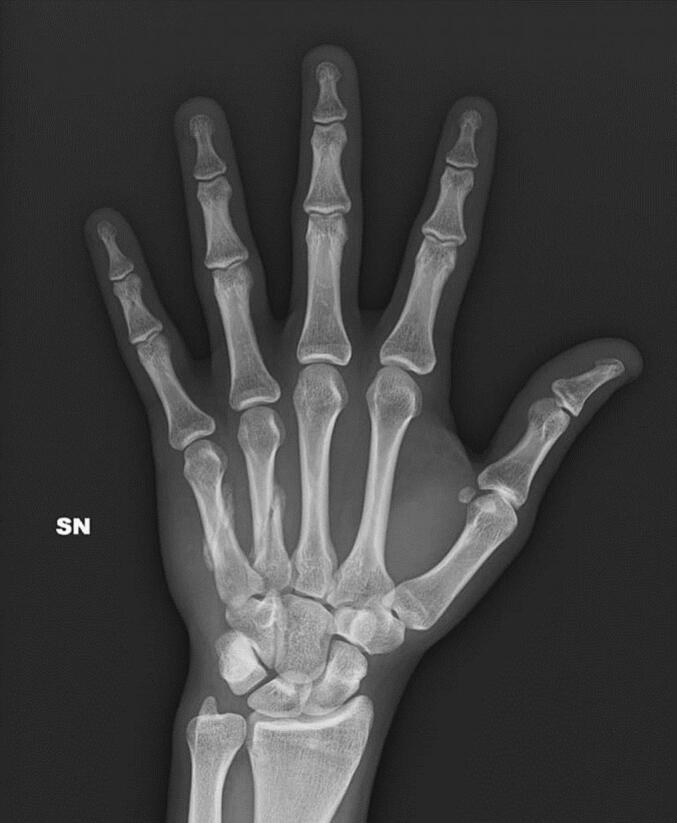
Static ulnar gutter brace orthosis.

The agreement was to perform a control radiograph after the first match to verify that the fractures had not moved. The subsequent week's control radiographs showed no displacement of the fracture stumps. The medical recommendation was then to continue the treatment until a 4-week post-trauma radiographic evaluation. Meanwhile, the player continued regular training and participation in championship matches. At the end of the predetermined 4 weeks, the patient underwent the final control radiographs (Fig. 3), which indicated that the radiographic findings were unchanged from the initial post-trauma radiographs. Due to a new thigh injury that required a period of rest, we could not re-evaluate the patient clinically.

### 3.2 Timeline

#### 3.2.1 Follow-up evaluation at 10 weeks post-injury

At 10 weeks post-injury, a detailed follow-up examination was conducted to assess the range of motion in the player's injured hand, focusing on the fourth and fifth digits which had sustained spiral diaphyseal fractures. The evaluation was performed using a goniometer, a standard instrument in orthopedic and rehabilitation settings for measuring joint angles.

Fourth Digit Assessment:

• Metacarpophalangeal (MP) Joint: The flexion at the metacarpophalangeal joint of the fourth digit was measured at 90°. This indicates a full range of motion, as normal flexion at the MP joint typically ranges between 85 and 100°.
• Proximal Interphalangeal (PIP) Joint: The fourth digit's proximal interphalangeal joint exhibited a flexion of 90°, which falls within the normal functional range (normally up to 100–110°).
• Distal Interphalangeal (DIP) Joint: Flexion at the distal interphalangeal joint was measured at 60°. While this is slightly below the average range for DIP joint flexion (which can be up to 70–80°), it represents significant functional recovery post-injury.

Fifth Digit Assessment:

• Metacarpophalangeal (MP) Joint: The fifth digit demonstrated 90 degrees of flexion at the MP joint, indicating a normal range of motion.
• Proximal Interphalangeal (PIP) Joint: An impressive flexion of 95° was recorded at the PIP joint, showcasing an excellent recovery and functional range.
• Distal Interphalangeal (DIP) Joint: The DIP joint flexion was measured at 63°. Similar to the fourth digit, this is slightly lower than the typical range but still represents a significant recovery considering the nature of the injury and treatment.

Throughout the 10-week recovery period, the soccer player actively participated in training sessions and competitive matches. This continuous involvement in high-level athletic activity was made possible by the effectiveness of the conservative treatment approach and the use of a specifically designed orthosis, which provided the necessary support and protection to the injured hand without significantly restricting mobility or function.

## 4 Discussion

The evolution of conservative treatment for metacarpal fractures over the years has progressed from the initial plaster casts of Burton to the brace and modified brace techniques. These methods have seen a gradual shortening of stabilization orthoses and a shift from plaster to thermomoldable materials. The shortened orthoses immobilize only the joints proximal and distal to the fracture, leaving the IP free. These joints are actively and immediately mobilized post-trauma, significantly reducing the risk of joint stiffness and tendinous adhesions. Like all patients undergoing this treatment pathway, the player began active flexion-extension mobilization of the proximal and distal interphalangeal joints (IFP and IFD), ensuring that by the end of the healing process, the only joints potentially experiencing temporary stiffness would be the MP. Empirical evidence suggests that such stiffness is quickly resolved through simple active flexion-extension movements. A study conducted on students in the United States also demonstrated that immobilization without trauma or surgical intervention drastically reduces the severity of stiffness, which resolves quickly, as seen in empirical experiences. The conservative treatment chosen by the team allowed the player to continue his professional activities with a moderate risk of fracture displacement, controlled by the stabilizing brace. We can confidently assert the successful healing of both fractures without further displacement. An additional advantage of this treatment was its minimal cost, represented by the thermoplastic material used for the brace. Furthermore, the brace's extreme lightness and compactness enabled the athlete to continue his competitive activities without interruption or limitation. However, a limitation of this study is the inability to re-evaluate the patient after the immobilization period to assess the degree of joint mobility and/or post-immobilization stiffness of the metacarpophalangeal joints.

## Informed consent

Informed consent has been obtained from all individuals included in this study.

## Ethical approval

Ethical approval is not a requirement at our institution for reporting individual cases or case series.

## Funding

Authors state no funding involved.

## Author contribution

RT and PB contributed to conception and design of the study; RT and DD to data acquisition, RT to data analysis and interpretation; RT and PB contributed to draft the manuscript; RT and FP contributed to the critical revision for important intellectual content. All authors read and approved the final version of the manuscript.

## Guarantor

Roberto Tedeschi.

## Conflict of interest statement

Authors state no conflict of interest.
